# Ideal Nasal Preferences: A Quantitative Investigation with 3D Imaging in the Iranian Population

**DOI:** 10.1055/a-2091-6820

**Published:** 2023-08-02

**Authors:** Kiarash Tavakoli, Amir K. Sazgar, Arman Hasanzade, Amir A. Sazgar

**Affiliations:** 1 Department of Otolaryngology, Head and Neck Surgery, Otorhinolaryngology Research Center, Tehran University of Medical Sciences, Tehran, Iran; 2 School of Medicine, Medical School, Shahid Beheshti University of Medical Sciences, Tehran, Iran

**Keywords:** esthetics, nose, image processing, computer-assisted, photography

## Abstract

**Background** Though in facial plastic surgery, the ideal nasal characteristics are defined by average European-American facial features known as neoclassical cannons, many ethnicities do not perceive these characteristics as suitable.

**Methods** To investigate the preferences for nasofrontal angle, nasolabial angle, dorsal height, alar width, and nasal tip projection, manipulated pictures of one male and one female model were shown to 203 volunteer patients from a tertiary university hospital's facial plastic clinic.

**Results** The most esthetically preferred nasofrontal angles were 137.64 ± 4.20 degrees for males and 133.55 ± 4.53 degrees for females. Acute nasofrontal angles were more desirable in participants aged 25 to 44. The most preferred nasolabial angles were 107.56 ± 5.20 degrees and 98.92 ± 4.88 degrees, respectively. Volunteers aged 19 to 24 preferred more acute male nasolabial angles. A straight dorsum was the most desirable in both genders (0.03 ± 0.78 and 0.26 ± 0.75 mm, respectively). The ideal male and female alar widths were –0.51 ± 2.26 and –1.09 ± 2.18 mm, respectively. More 45- to 64-year-old volunteers preferred alar widths equal to intercanthal distance. The ideal female and male tip projections were 0.57 ± 0.01 and 0.56 ± 0.01, respectively.

**Conclusion** Results indicate that the general Iranian patients prefer thinner female noses with wider nasofrontal angles for both genders. However, the ideal nasolabial angles, dorsal heights, and tip projections were consistent with the neoclassical cannons. Besides ethnic differences, the trend of nasal beauty is also affected by gender, age, and prior history of esthetic surgery.

## Introduction

An esthetic nose plays an important role in the definition of a face's beauty. This is not only due to its central location on the face, but also because it is among the first facial features that draw an observer's attention. 1 2 3 From ancient times, humans have sought to define the ideal nasal parameters, resulting in the neoclassical cannons that defined the ideal nose from average European-American facial features under the influence of ancient Greek and Renaissance findings. 4 5

Although plastic surgeons use neoclassical cannons as a goal in their procedures, they are not desirable for all ethnicities. 2 3 6 7 8 These inconsistencies highlight an insufficiency in the concept of universally ideal nasal characteristics and point to the importance of regional research so that plastic surgeons may reach satisfactory results in different societies.

Despite many studies trying to find ideal nasal characteristics using two-dimensional (2D) pictures in different ethnicities, 1 3 6 7 9 this format is not as practical as three-dimensional (3D) imaging to represent body part characteristics such as nasal features alongside other facial subunits. 8

To our knowledge, this is one of the first studies evaluating ideal nose shapes using 3D technology in the Middle East. The purpose of our study is to evaluate viewer opinions on the esthetic nose using 3D imaging with specific focus on five parameters, including nasofrontal angle (NFA), nasolabial angle (NLA), dorsal height (DH), alar width (AW), and tip projection (TP).

## Methods




**Supplementary Video S2**


This cross-sectional study was authorized by the Institutional Review Board, and its execution adhered to the ethical principles set forth in the Declaration of Helsinki regarding human subject research. Written consent was obtained from the two selected models (one male and one female) in the facial plastic clinic of a tertiary university hospital from 2020 to 2021, allowing their images to be used for research and publication purposes. The inclusion criteria for male and female models in the study were Iranian ethnicity, aged between 20 and 29, body mass index between 18 and 24.9, absence of craniofacial deformity (such as cleft nose), and no previous history of esthetic surgery (such as rhinoplasty) or significant facial trauma. The subjects were photographed using a Vectra H1 (Canfield Scientific, Parsippany, NJ). The Mirror software (Canfield Scientific, Inc., NJ) and the Vectra Analysis Module (Canfield Scientific, Inc., Fairfield, NJ) were used respectively to measure and modify to desired scales the NFA, NLA, TP, AW, and DH of the 3D photographs of the models.

**Fig. 1:**
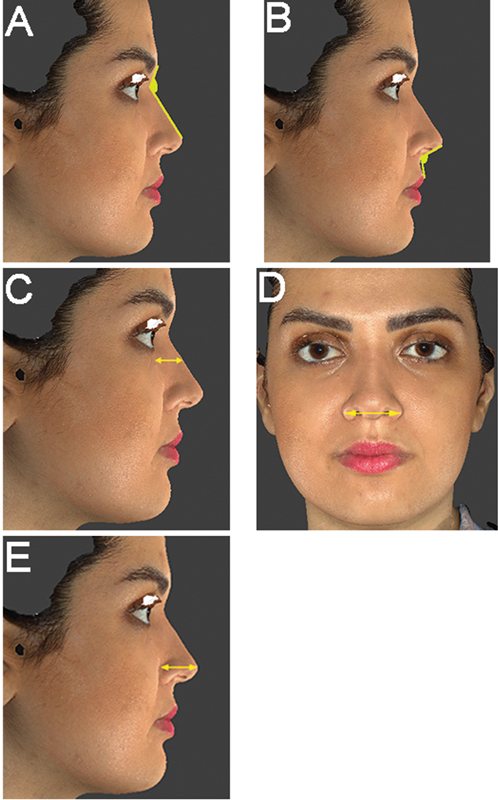
Five different parameters were modified in the selected female subject including ( **A** ) nasofrontal angle (NFA), ( **B** ) nasolabial angle (NLA), ( **C** ) dorsal height (DH), ( **D** ) alar width (AW), and ( **E** ) tip projection (TP).

The NFA is defined as the angle formed by the intersection of the line drawn from the nasion tangential to the superior surface of the nose and the line from the soft tissue glabella to the nasion ( Fig. 1A ). The NLA is defined as the angle formed by the intersection of the lines tangent to the labial surface of the upper lip on the lateral view and the inferior border of the nose ( Fig. 1B ). We also described the level of the nasal dorsum in relation to a line connecting the nasal tip-defining points to the radix ( Fig. 1C ). The distance between the left and right ala was defined as AW ( Fig. 1D ). The nasal TP was described as the ratio of the length of the line from the alar crease to the nasal tip that is perpendicular to the line tangent to the alar crease divided by the length of the line from the nasion to the nasal tip ( Fig. 1E ).

NFAs of 125, 130, 135, 140, and 145 degrees were chosen for both male and female noses. NLAs of 95, 100, 105, 110, 115, and 120 degrees were selected for the female model, and NLAs of 85, 90, 95, 100, and 105 degrees were chosen for the male model. The DHs selected for the female nose included a straight dorsum, 1- and 2-mm concave dorsums, and a 1-mm convex dorsum in comparison to the straight nasal dorsum in the female model. The DHs selected for the male nose included a straight dorsum, 1- and 2-mm concave dorsums, and 1- and 2-mm convex dorsums compared with the straight nasal dorsum in the male model. The AWs selected for the male and female noses consist of a width equal to the intercanthal distance, widths 2 and 4 mm narrower than the intercanthal distance, and widths 2 and 4 mm wider than the intercanthal distance. For TP, the ratios of 0.55, 0.57, and 0.59 were selected for both male and female noses. By changing only one of the nasal parameters in each picture, a total of 46 3D faces were generated. The 3D faces were merged to make a single 21-second length video clip for each nasal parameter.

The required sample size for the study was determined using G*Power (version 3.1.9.2 for Windows, Heinrich-Heine-Universität Düsseldorf, Düsseldorf, Germany) with analysis of variance, which resulted in 200 participants with a margin of error of 5% and power of 80%. A two-part questionnaire was developed based on the videos and subsequently filled out by patients willing to participate in the study ( Supplementary Data S1 ). The volunteers were asked to sit at a desirable distance from a 20-inch monitor and select the most esthetically pleasing option from the displayed alternatives ( Supplementary Video S2 ). No time limitation was placed on the questions, and the videos were replayed until participants chose an answer. The volunteers were also asked about their age, sex, education, residency, and prior history of esthetic surgery. Based on the Medical Subject Headings keywords, age groups were divided into adolescent, young adult, adult, middle-aged, and aged. Any incomplete questionnaires were excluded, and the related answers were not considered in the study results. Non-Iranian participants living in Iran and Iranians who had lived abroad were excluded to reduce confounding cultural effects on responses.

All data were collected and analyzed anonymously using IBM SPSS Statistics for Mac, version 16 (IBM Corp., Armonk, NY). The chi-square and Fisher's exact tests were used to compare categorical values, while the *t* -test was used to compare numeric values. Two-sided *p* -values of less than 0.05 were considered statistically significant.

## Results

A total of 203 volunteers partook in the study. The majority were female (79.8%), 25 to 44 years old (65.5%) with a mean age of 31.2 ± 9.9, had a bachelor's degree (38.4%), and a history of esthetic surgery (59.6%). Participants were from 35 different cities in Iran, but most of them lived in Tehran (63.5%; Table 1 ).

**Table 1:** Demographic characteristics of the population

Demographic characteristics	*N* (%)
Age	
Adolescent (< 18 y)	13 (6.4)
Young adult (19–24 y)	39 (19.2)
Adult (25–44 y)	133 (65.5)
Middle aged (45–64 y)	17 (8.4)
Aged (> 65 y)	1 (0.5)
Gender	
Male	41 (20.2)
Female	162 (79.8)
Residency	
Tehran	129 (63.5)
Non-Tehran	74 (36.5)
Education	
High school	9 (4.4)
Diploma	61 (30.1)
Bachelor's degree	78 (38.4)
Master of science	33 (16.3)
PhD (Doctor of Philosophy)	22 (10.8)
Previous esthetic surgery	
Yes	121 (59.6)
No	82 (40.4)

### Nasofrontal Angle

**Fig. 2:**
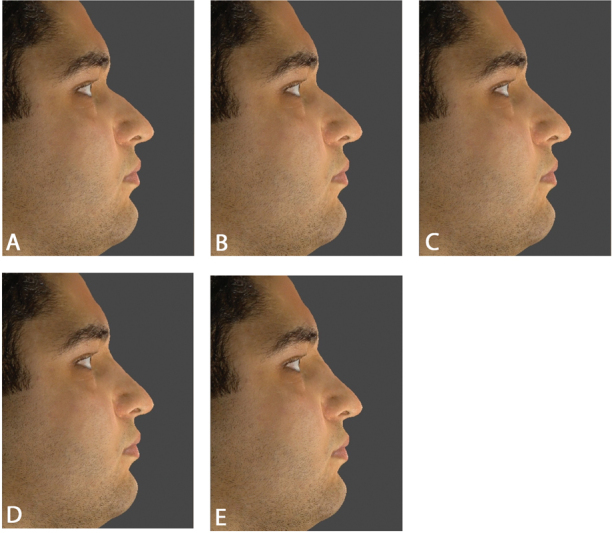
The nasofrontal angle (NFA) was modified to 125, 130, 135, 140, and 145 degrees in the selected male subject.

**Fig. 3:**
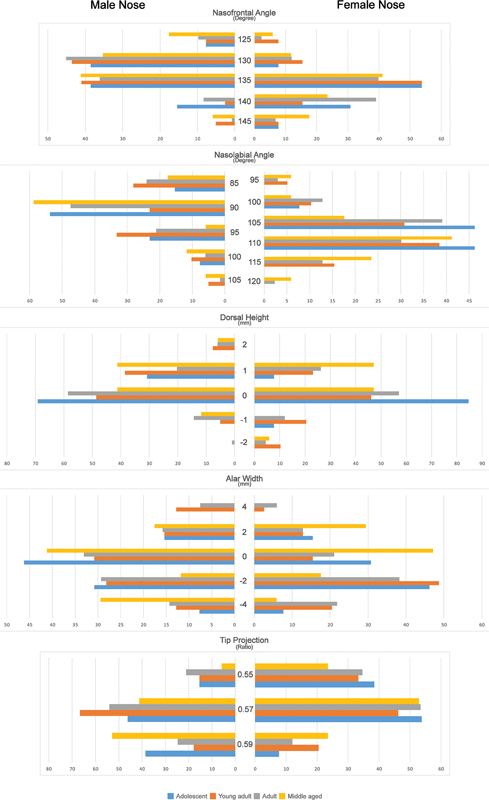
Distribution of participant opinions regarding nasofrontal angle (NFA), nasolabial angle (NLA), dorsal height (DH), alar width (AW), and tip projection (TP) based on age group.

For all volunteers in this study, 133.55 ± 4.53 degrees was the most esthetic female NFA with a median and mode of 135 degrees. The most preferred female NFA was 135 degrees (43.8%), while only a small portion of participants considered 145 degrees esthetic for the female NFA (3.4%). The most esthetic male NFA was 137.64 ± 4.20 degrees among all participants with a median and mode of 140 degrees ( Fig. 2 ). Of the participants, 43.8% chose 140 degrees as the most esthetic male NFA. The least esthetic male NFA was 125 degrees with only 2% of volunteers selecting it. Age was found to have a significant impact on participant perception of the most esthetic female NFA. Volunteers aged between 25 and 44 years preferred more acute NFAs for females than comparators (39.1% vs. 20% for 130 degrees, *p* -value = 0.051). On the other hand, young adult participants preferred acute female NFAs less than older age groups (15.4% vs. 36.6% for 130 degrees, *p* -value = 0.050). There was no statistically significant difference among the age subgroups about male NFAs or the residency subgroups about female and male NFAs ( *p* -value = 0.057 and 0.307, respectively; Fig. 3 and Table 2 ).

**Table 2:** Distribution and p-values of participant opinions regarding nasofrontal angle, nasolabial angle, dorsal height, alar width, and tip projection based on gender, residency, and history of previous esthetic facial surgery

			Female nose						Male nose						
Nasofrontal angle	Angle (degree)		125		130	135	140	145	*p* -Value	125	130	135	140	145	*p* -Value
	Gender	Female	12 (7.4)		52 (32.1)	73 (45.1)	21 (13.0)	4 (2.5)	0.565	2 (1.2)	12 (7.4)	61 (37.7)	70 (43.2)	17 (10.5)	0.583
		Male	4 (9.8)		14 (34.1)	16 (39.0)	4 (9.8)	3 (7.3)		2 (4.9)	2 (4.9)	15 (36.6)	19 (46.3)	3 (7.3)	
	Previous esthetic surgery	Yes	8 (6.6)		41 (33.9)	51 (42.1)	15 (12.4)	6 (5.0)	0.576	1 (0.8)	8 (6.6)	45 (37.2)	53 (43.8)	14 (11.6)	0.595
		No	8 (9.8)		25 (30.5)	38 (46.3)	10 (12.2)	1 (1.2)		3 (3.7)	6 (7.3)	31 (37.8)	36 (43.9)	6 (7.3)	
Nasolabial angle	Angle (degree)		95	100	105	110	115	120	*p* -Value	85	90	95	100	105	*p* -Value
	Gender	Female	3 (1.9)	15 (9.3)	56 (34.6)	62 (38.3)	20 (12.3)	6 (3.7)	0.021	4 (2.5)	10 (6.2)	33 (20.4)	75 (46.3)	40 (24.7)	0.205
		Male	1 (2.4)	13 (31.7)	12 (29.3)	11 (26.8)	3 (7.3)	1 (2.4)		1 (2.4)	6 (14.6)	12 (29.3)	14 (34.1)	8 (19.5)	
	Previous esthetic surgery	Yes	3 (2.5)	15 (12.4)	39 (32.3)	42 (34.7)	18 (14.9)	4 (3.3)	0.477	4 (3.3)	6 (5.0)	27 (22.3)	55 (45.5)	29 (24.0)	0.366
		No	1 (1.2)	13 (15.9)	29 (35.4)	31 (37.8)	5 (6.1)	3 (3.7)		1 (1.2)	10 (12.2)	18 (22.0)	34 (41.5)	19 (23.2)	
Dorsal height	Height (mm)		–2			–1	0	1	*p* -Value	–2	–1	0	1	2	*p* -Value
	Gender	Female	9 (5.6)			18 (11.1)	94 (58.0)	41 (25.3)	0.528	1 (0.6)	20 (12.3)	90 (55.6)	41 (25.3)	10 (6.2)	0.883
		Male	2 (4.9)			7 (17.1)	19 (46.3)	13 (31.7)		0 (0.0)	3 (7.3)	24 (58.5)	12 (29.3)	2 (4.9)	
	Previous esthetic surgery	Yes	6 (5.0)			17 (14.0)	63 (52.1)	35 (28.9)	0.541	1 (0.8)	5 (7.3)	52 (63.4)	23 (28.0)	1 (1.2)	0.037
		No	5 (6.1)			8 (9.8)	50 (61.0)	19 (23.2)		0 (0.0)	17 (14.0)	62 (51.2)	30 (24.8)	11 (9.1)	
Alar width	Width (mm)		–4		–2	0	2	4	*p* -Value	–4	–2	0	2	4	*p* -Value
	Gender	Female	33 (20.4)		62 (38.3)	36 (22.2)	25 (15.4)	6 (3.7)	0.638	24 (14.8)	49 (30.2)	54 (33.3)	26 (16.0)	9 (5.6)	0.101
		Male	6 (14.6)		18 (43.9)	10 (24.4)	4 (9.8)	3 (7.3)		6 (14.6)	7 (17.1)	15 (36.6)	6 (14.6)	7 (17.1)	
	Previous esthetic surgery	Yes	33 (27.3)		42 (34.7)	22 (18.2)	18 (14.9)	6 (5.0)	0.005	23 (19.0)	26 (21.5)	45 (37.2)	18 (14.9)	9 (7.4)	0.067
		No	6 (7.3)		38 (46.3)	24 (29.3)	11 (13.4)	2 (3.7)		7 (8.5)	30 (36.6)	24 (29.3)	14 (17.1)	7 (8.5)	
Tip projection	Ratio		0.55		0.57		0.59		*p* -Value	0.55	0.57		0.59	*p* -Value	
	Gender	Female	23 (14.2)		86 (53.1)		53 (32.7)		0.913	41 (25.3)	92 (56.8)		29 (17.9)	0.607	
		Male	6 (14.6)		20 (48.8)		15 (36.6)			13 (32.5)	20 (48.8)		8 (19.5)		
	Previous esthetic surgery	Yes	17 (14.0)		59 (48.8)		45 (37.2)		0.384	30 (24.8)	65 (53.7)		26 (21.5)	0.328	
		No	12 (14.6)		47 (57.3)		23 (28.0)			24 (29.3)	47 (57.3)		11 (13.4)		

### Nasolabial Angle

For the study population, 107.56 ± 5.20 degrees was the most preferred female NLA with a median and mode of 110 degrees. The most esthetic choice for female NLA was 110 degrees (36.0%), while 95 degrees was represented as the least esthetic female NLA with only 2.0% of the population selecting it. In this study, 98.92 ± 4.88 degrees was the most esthetic male NLA with a median and mode of 100 degrees. Of the participants, 43.8% chose 100 degrees as the most esthetic male NLA. On the other hand, 85 degrees was the least esthetic male NLA with only 2.5% choosing it. Gender statistically affected participant opinions about the most esthetic female NLA. Male volunteers preferred more acute female NLAs than female comparators (31.7% vs. 9.3% for 100 degrees, *p* -value = 0.021). We did not find a statistically significant difference between male and female opinions about the most esthetic male NLA. Age also played an important role in presumptions about male NLAs. The young adult group preferred more acute male NLAs compared with other participants (33.3% vs. 19.5% for 95 degrees, *p* -value = 0.024). There was no statistically significant difference between the age subgroups for female NLAs or the residency subgroups about female and male NLAs ( *p* -value = 0.485 and 0.383, respectively; Fig. 3 and Table 2 ).

### Dorsal Height

The study findings indicate that the most desirable female DH was 0.03 ± 0.78 mm, with a median and mode of 0 mm. Over half of the participants (55.7%) favored a straight dorsum (0 mm) over other options, while the least acceptable choices were concave DHs, with only 5.4% of volunteers choosing –2 mm. The most preferred male DH in this study was 0.26 ± 0.75 mm, with a median and mode of 0 mm. The majority of the population (56.2%) chose a straight dorsum over the other options. A male DH within –2 mm from the base was the least desirable choice, with only 0.5% of the population choosing it. Patients with a prior history of esthetic surgery preferred a straight dorsum for the male nose more frequently than comparators (63.4% vs. 51.4%, *p* -value = 0.037). There was no statistically significant difference between residency subgroups regarding female and male DHs ( *p* -value = 0.335 and 0.644, respectively; Fig. 3 and Table 2 ).

### Alar Width

In this study, the ideal female AW was –1.09 ± 2.18 mm, with a median and mode of –2 mm. Of the population, 39.4% selected the female nose that was 2 mm narrower than the intercanthal distance. AWs wider than the intercanthal distance were least desirable. Only 14.3% of the population chose the AW 2 mm wider than the intercanthal distance, while 4.4% chose the AW 4 mm wider than the intercanthal distance. The ideal male AW was –0.51 ± 2.26 mm with a median and mode of 0 mm. Among the volunteers, 34% chose the male nose with an AW equal to the intercanthal distance. Like the female nose, AWs wider than the intercanthal distance were the least desirable male features. Age differences played an important role in participant opinion on the ideal female AW, with a higher percentage of middle-aged volunteers preferring the AW equal to the intercanthal distance compared with the other age groups (47.1 vs. 20.4, *p* -value = 0.015). There was no statistically significant difference between residency subgroups regarding the preferences for female and male AWs ( *p* -value = 0.474 and 0.802, respectively; Fig. 3 and Table 2 ).

### Tip Projection

The results showed that the ideal female TP is 0.57 ± 0.01, with a median and mode of 0.57. More than half of the participants (52.2%) chose this ratio as the most esthetic among the various options, while only a small percentage (14.3%) favored a ratio of 0.55. The most esthetic male TP was 0.56 ± 0.01, with a median and mode of 0.57. The majority of the population (55.2%) chose 0.57 as the most esthetic feature for the male TP, while only 18.2% of the population chose 0.59 as the most esthetic. There was no statistically significant difference in the opinions of the residency subgroups about female and male TPs ( *p* -value = 0.077 and 0.664, respectively; Fig. 3 and Table 2 ).

## Discussion

The present study investigated the preferred nasal characteristics among Iranian patients. Our findings demonstrated that the perception of nasal beauty is influenced by various factors, including ethnicity, gender, age, and prior history of esthetic surgery. A successful rhinoplasty procedure relies on not only a functional and esthetic nose but also the satisfaction of the patient.

Although the neoclassical canons have been widely used as the standard for esthetic parameters in rhinoplasty, the literature shows that the textbook ideal proportions may not be acceptable for all ethnicities, 4 10 making research into the desirable facial characteristics of people in different regions important. 11 12 The emergence of 3D imaging as a novel technology has provided a great opportunity to assess the ideal nasal parameters of different ethnicities by overcoming the shortcomings of 2D imaging. 13 14 Despite rhinoplasty being one of the most common plastic surgery procedures in Middle Eastern countries, 15 there is a paucity of data on the ideal nasal characteristics for people from this region. To our knowledge, this study is among the first to utilize 3D imaging technology to identify the ideal nasal profile of the general population in the Middle East.

Based on the study conducted by Powell and Humphreys, the ideal NFA varies between 115 and 130 degrees. 16 However, our findings do not conform to neoclassical cannons by showing that Iranian patients may prefer more obtuse NFAs. In line with our findings, Mafi et al have also demonstrated wider NFAs to be more acceptable in Iran. 12 The influence of ethnicity on the ideal NFA is further supported by Yu et al research conducted on Korean patients, which found that they preferred wider NFAs for both males and females than the neoclassical facial standards. 17 These differences in preference can be attributed to morphological variations among different ethnic groups. For example, Iranian patients have a straighter frontonasal area, which can affect the position of the glabella, one of the key positions in NFA measurement. 12

The established articles define the ideal NLAs of 103 to 108 degrees for females and 95 to 100 degrees for males, 18 19 20 and our study indicates that the ideal NLAs for Iranian patients are consistent with these neoclassical cannons. The results of Biller and Kim agree with ours in that the ethnicities of the model and voters do not affect the preferred NLA, and that younger participants tend to prefer more acute NLAs than older volunteers. 21 The outcomes of research by Sinno et al validate our own discoveries, showing that a female NLA range of 104.9 ± 4.0 degrees is ideal, while for males, the ideal is 97.0 ± 6.3 degrees. No statistically significant differences were observed among gender and age cohorts. 6 On the other hand, the results of Alharethy and Armijo et al are inconsistent with ours by showing more acute ideal male and female NLAs than what is reported in the literature. 10 22 Variations in methods used for NLA measurement may be the main reason for differences in nasolabial values and thus divergences in outcomes. Leach recommended measuring the NLA as the angle between the long axis of the nostril and the line perpendicular to the Frankfurt horizontal plane. 23 Although this method is a better way of measuring NLA in faces with procumbent incisors or protruding maxillae, the Frankfort horizontal line is less accurate on soft tissues. 21 Due to this shortcoming, determining the NLA as the angle between the labial surface of the upper lip and the inferior border of the nose on the lateral view is not only the most common method in studies with esthetic purposes but also a suitable method in 3D imaging technologies such as Vectra. 24

Our study shows that a straight nasal dorsum is the most preferred form for both male and female noses. This conclusion is supported by Alharethy, who revealed that a straight nasal dorsum is the most desirable nasal profile in the Middle Eastern region, and that a posterior nasal dorsum within 2 mm is the most approved male nasal dorsum. 25 The findings of a study conducted by Yu et al also corroborate ours by showing that a straight dorsum is the most acceptable nasal form among both males and females. 17 The neoclassical cannons suggest that the most esthetically pleasing AW is equal to the intercanthal distance, 26 but we found that this ideal does not hold true for the female nose in the Middle Eastern region. Participant age differences can affect perceptions about the most desirable AW, as older participants tend to prefer AWs equal to the intercanthal distance, unlike younger ones. Many published articles have proposed various ratios for the most esthetic nasal TPs. 27 28 However, Devcic et al showed that the most esthetic faces have TPs that are close to the Goode ratio. 9 Mohebbi et al also demonstrated that the Goode ratio is the most preferred among the general Iranian populace. 29 Our results show that a TP ratio of 0.57 is the most preferred for both male and female noses within the range proposed by the Goode ratio and that deviation from 0.57 decrease the perceived esthetics of the TP.

To our knowledge, this is one of the first reports leveraging 3D imaging technology to evaluate the ideal nasal profile of the general population in the Middle East. Even though the data were collected in a single city in Iran (Tehran), the study population comprised participants from different cities in Iran with various genders, age subgroups, and educational levels. While the sample size cannot show the Iranian society's perspective about the ideal nose, it can help the researcher understand the differences in the conception of beauty among various societies and ethnicities.

When interpreting the results of our current analysis, several limitations should be taken into account. First, although the pictures were manipulated to have typical characteristics of the Iranian profile, the impact of each nasal characteristic on other features was not considered. Second, some patients had difficulty discerning the differences between AWs, which makes AW calculations susceptible to biases. Third, some patients were unwilling to participate in the study, which may have introduced biases.

Our study shows that the general Middle Eastern population prefers thinner female noses with wider NFAs for both genders. The ideal NLA, DH, and TP did not depart from the ideal characteristics of neoclassical cannons. Furthermore, the perception of nasal esthetics is affected by factors such as gender, age, and prior history of esthetic surgery.
