# Supplementary material for: Ideal Nasal Preferences: A Quantitative Investigation with 3D Imaging in the Iranian Population
Source: Arch Plast Surg. 2023 Aug 2;50(4):340–7. doi: 10.1055/a-2091-6820 (PMC10411228; doi:10.1055/a-2091-6820)
Supplement: Supplementary file 2 — Supplementary Material [file 10-1055-a-2091-6820-s22dec0222oa.pdf]

Ideal nasal preferences: A quantitative investigation with 3D imaging in the Iranian population

Kiarash Tavakoli MS, Amir K. Sazgar* MS, Arman Hasanzade MS, Amir A. Sazgar MD

Tehran University of Medical Sciences

***ak-sazgar@student.tums.ac.ir**

Greetings and Regards

Thank you very much for giving us your time and cooperating in this research.
In this project, we want to know your opinion about the ideal nose. Your information will be used anonymously. After filling in the information of the first section, complete the second section by watching the videos.

First Section

- **Age:**
- **Sex:** Female ☐ Male ☐
- **City of residence :**
- **Job :**

- **Education:** Under diploma ☐ Diploma ☐ Bachelor's degree ☐ Master's degree ☐ Doctoral degree and higher ☐

- **Have you ever had a cosmetic surgery?** Yes ☐ No ☐
- **What surgery have you done?**

Second Section

In the next 10 minutes, 10 short videos will be played for you.
Please, in each section, according to the description of the video, choose the best image in your opinion and mark it below.

	1	2	3	4	5	6
**First part**						
1						
2						
3						
4						
5						
**Second part**						
1						
2						
3						
4						
5						

"Thank you for your time and patience"
